# Effect of Metabolic Stress to High-Load Exercise on Muscle Damage, Inflammatory and Hormonal Responses

**DOI:** 10.3390/sports13040111

**Published:** 2025-04-09

**Authors:** Séverine Stragier, Jacques Duchateau, Frédéric Cotton, Julie Smet, Fleur Wolff, Jérémy Tresnie, Alain Carpentier

**Affiliations:** 1 Research Unit in Cardio-Respiratory Physiology, Exercise & Nutrition, Faculty of Human Movement Sciences, Université libre de Bruxelles, 1070 Brussels, Belgium; severine.stragier@ulb.be; 2 Laboratory of Applied Biology and Neurophysiology, Faculty of Human Movement Sciences, Université libre de Bruxelles, 1070 Brussels, Belgium; jacques.duchateau@ulb.be; 3 Laboratoire Hospitalier Universitaire de Bruxelles, Clinical Chemistry, Université libre de Bruxelles, 1070 Brussels, Belgium; frederic.cotton@lhub-ulb.be (F.C.); fleur.wolff@lhub-ulb.be (F.W.); 4 Laboratoire Hospitalier Universitaire de Bruxelles, Immunology, Université libre de Bruxelles, 1070 Brussels, Belgium; julie.smet@lhub-ulb.be (J.S.); jeremy.tresnie@lhub-ulb.be (J.T.)

**Keywords:** strength training methods, muscle regeneration, lactate, blood biomarkers

## Abstract

To assess the impact of metabolic stress on blood lactate, muscle damage, inflammatory and hormonal responses following a high-load (70% maximum) strength training session, we compared two methods with a similar number of repetitions but that differed by their metabolic demand: the 3/7 method consisting in two series of five sets of an increasing number of repetitions (3 to 7) with a short inter-set interval (15 s) and the 8 × 6 method that comprises eight sets of six repetitions with a longer inter-set interval (2.5 min). Blood concentrations in lactate, creatine kinase (CK), myoglobin (MB), interleukine-6 (IL-6), leukocytes, growth hormone (GH), insulin-like growth factor-1 (IGF-1) and cortisol were determined before and after each session. Lactate concentration increased more (11.9 vs. 3.1 mmol/L; *p* < 0.001) for the 3/7 method whereas CK and MB concentrations were augmented similarly (*p* > 0.05) for both methods. Inflammatory markers (leukocytes and IL-6) increased (*p* < 0.01) more after the 3/7 method. GH and cortisol concentrations also increased more (*p* < 0.001) after the 3/7 method with no difference in IGF-1 concentrations between methods. Positive associations were found between the change in lactate and changes in IL-6 (r^2^ = 0.47; *p* < 0.01), GH (r^2^ = 0.58; *p* < 0.001) and cortisol (r^2^ = 0.61; *p* < 0.001) concentrations. In conclusion, the greater lactate accumulation induced by short inter-set intervals during a high-load training session is associated with enhanced inflammatory and hormonal responses, suggesting that metabolic stress might contribute to the greater adaptative response previously observed with this method.

## 1. Introduction

Although the adaptations to strength training have been widely studied, the mechanisms triggering the development of muscle mass remain a matter of debate [1,2,3,4].

For many years, it has been considered that a high level of muscle tension (i.e., mechanical tension) associated with acute anabolic hormonal production at the muscle level [i.e., insulin-like growth factor (IGF-1)] were key factors to stimulate protein synthesis and to incorporate satellite cells into muscle fibers, leading to muscle hypertrophy [5,6]. However, in the last two decades, many studies have shown that a training program using low loads (20–50% of one repetition maximal—1 RM) mobilized under reduced muscle perfusion, induced by the application of an external pressure (inflated cuff, tourniquet), can also increase muscle mass [7,8,9,10]. Such an ischemic condition reduces blood flow in the involved muscles and in turn the oxygen supply, which produces a marked accumulation of metabolite by-products, such as lactate, hydrogen ions (H^+^), inorganic phosphate (Pi) and others [11,12]. Among these metabolites, lactate, which is a key marker of metabolic stress, may have an anabolic role [13] as a signaling molecule that promotes myogenic differentiation of satellite cells [14].

In addition, the use of high-load exercise, notably eccentric contractions, may further be associated with microtraumas [15] resulting in the release of intracellular proteins [myoglobin (MB) and creatine kinase (CK)] in blood circulation due to the disruption of the extracellular matrix and basal lamina of the sarcolemma [16]. In response to these microtraumas, a transient elevation in circulating concentrations of inflammatory-related markers such as interleukin-6 (IL-6; [17]) and leukocytes [18] has been reported. This inflammatory response appears to play a role in muscle regeneration after exercise-induced muscle damage [19,20], a process that seems to be amplified by metabolic stress [21,22,23].

Together, these observations suggest that both mechanical tension and metabolic stress may contribute to increase protein synthesis and, thereby, muscle repair and hypertrophy [1,24]. Training with blood flow occlusion presents some constraints [8] and does not appear to optimize the development of the neural aspects of muscle strength [3]; an alternative training condition to generate high metabolic stress can be accomplished by using moderate to high-load exercises with briefs inter-set rest intervals. In this context, we recently underscored the efficacy of a training method (called the 3/7 method; [25]) using a load of 70% of 1 RM (mechanical stimulus) and a very brief rest interval (15 s) between sets (metabolic stimulus) to promote strength gains and muscle hypertrophy [26]. The superiority of this method over a more classical method (eight sets of six repetitions—8 × 6 method), using a similar load and number of repetitions but a much longer inter-set interval (2.5 min), may be the consequence of a greater metabolic stimulus induced by the brief inter-set rest interval in the 3/7 method, as supported by a greater deficit in muscle oxygenation tested by near-infrared spectroscopy [27].

Therefore, to improve our understanding of the interactions between metabolic stress and muscle damage, inflammatory and hormonal blood markers, and thereby evaluate their potential role in triggering muscle regeneration and growth, we compared, in moderately trained young men, the acute effects of a training session comprising either the 3/7 or 8 × 6 method. We hypothesized that the 3/7 method would produce greater blood lactate accumulation compared with the 8 × 6 method, leading to an increased anabolic myokines response and a greater systemic hormonal production.

## 2. Materials and Methods

### 2.1. Subjects

Ten young men (23 ± 3 years, 84.2 ± 6.7 kg, 1.84 ± 0.06 m) participated in this study. All subjects signed an informed consent and underwent a medical check-up prior to their participation in the study. Individuals with orthopedics problems were excluded from this study. All procedures conformed to the Declaration of Helsinki.

**Figure 1:**
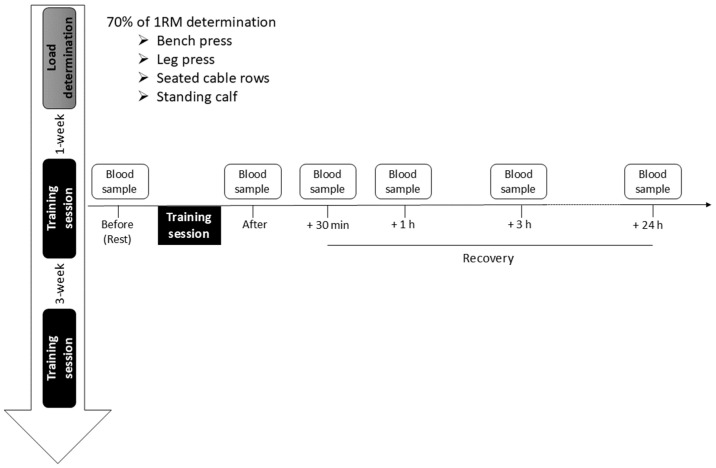
Experimental design organized in 3 sessions (load determination, first and second training session). Blood samples were collected before each training session, immediately after (After) and at 30 min (+30 min), 1 h (+1 h), 3 h (+3 h) and 24 h (+24 h) of recovery.

All subjects had recreational experience of strength training but were not engaged in any systematic strength training program for at least 6 months prior to their participation in the study. Each subject participated in 3 sessions, one familiarization session and two training sessions. The familiarization session served to accustom the subjects to the training exercises and to determine the training load. In each of the two training sessions, subjects performed, in a counterbalanced order, either the 3/7 method or the 8 × 6 method. These two training sessions were separated by at least 3 weeks (Figure 1). The participants were instructed to maintain their habitual physical activity during the entire duration of the study and to avoid the consumption of alcohol for 48 h and caffeine 12 h before each session. The day of the training sessions, they were asked to take a light lunch (2 to 3 slices of bread, with fat-free ham, and water).

### 2.2. Experimental Protocol and Determination of the Training Load

The two training sessions were conducted at the same time of the day for each subject and began about 30 min after the pre-exercise blood sample collection. Each training session was composed of 4 exercises whose order was the same: bench press, leg press, seated cable rows and standing calf raise. A rest period of 3 min was given between exercises. Each session began with a standardized warm-up of ~20 min, including running on a treadmill (7 min at 10–12 km/h), mobilizations of the mains joints of the upper and lower body and performing a single set of 6 repetitions of each exercise with a load of 70% of 1 RM. The 3/7 method consisted of 5 successive sets performed with an increment of 1 repetition per set (i.e., 1 set of 3 reps, 1 set of 4 reps, 1 set of 5 reps, 1 set of 6 reps and 1 set of 7 reps) and a rest interval of 15 s between two successive sets [25,26]. Two bouts of these 5 sets, separated by 2.5 min of rest, were performed. The 8 × 6 method consisted of 8 sets of 6 repetitions with a rest interval of 2.5 min between sets.

The load used during the training session corresponded to the load with which each subject was able to perform between 10 and 12 repetitions (~70% of 1 RM). This load was extrapolated from a 3-repetitions maximum (3RM) procedure, which corresponds approximately to a load of 90% of 1 RM [28]. The load was gradually increased by a minimum increment of 2.5 kg for the bench press, 10 kg for the leg press, 1.5 kg for the seated cable rows and 9 kg for the standing calf raise. The recovery between trials lasted at least 3 min. Three to 6 sets were necessary to determine the 3 RM. To check that this load corresponded to the expected number of repetitions (between 10–12), subjects performed a set during which the maximal number of repetitions was recorded [26]. If necessary, the training load was readjusted. For both methods, when the subject was unable to perform the prescribed numbers of repetitions during the training sessions, the supervisor provided the minimum amount of assistance necessary to help the subject to complete the required number of repetitions.

### 2.3. Blood Collection

Blood samples were collected from the basilic or cephalic forearm vein at the same time of the day during the two training sessions. The first blood sample was always taken between 7:00 and 8:30. Blood samples were taken before the warm-up period and after the training session (~5 min after the last exercise), and 30 min (+30 min), one hour (+1 h), three hours (+3 h) and twenty-four hours (+24 h) after the training session (Figure 1). Blood samples were collected in a 2 mL K2EDTA tube (BD Vacutainer^®^ from Becton Dickinson; Erembodegem, Belgium) for leukocytes, in a 4 mL lithium heparin tube (BD Vacutainer^®^) for CK and MB, in a 2 mL tube with NaF and Na2EDTA (BD Vacutainer^®^) for lactate and in three 3 mL tubes with an inert, stable gel (SSTTM II Advance BD Vacutainer^®^) to measure IL-6 and hormonal response. For IL-6, serum aliquots were immediately stored at −20 °C after centrifugation for later analysis. For GH and IGF-1, a dry tube sample was taken, followed by centrifugation, separation of the serum within 6 h and then freezing at −20 °C for later analysis. For the other biochemical parameters, the analyses were carried out on fresh plasma/serum.

### 2.4. Biochemical Analyses

Blood lactate and CK levels were quantified with a photometric method from Roche Diagnostics (Vilvoorde, Belgium) on Cobas analyzers. For IL-6 determination, each sample was assayed in duplicate by quantitative sandwich ELISA according to the manufacturer’s instructions (Quantikine immune assay kit, R&D Systems, Minneapolis, MN, USA). Circulating concentrations of cortisol and MB were measured using an electrochemiluminescence immunoassay from Roche Diagnostics (Vilvoorde, Belgium) on Cobas analyzers. IGF-1 and GH concentrations were assessed with a sandwich chemiluminescence immunoassay on the Liaison XL platform from Diasorin (Stillwater, MN, USA). Hematological parameters were obtained on a Unicel DxH800 from Beckman-Coulter (Analis, Suarlé, Belgium). Blood variables were corrected using the percentage change in hemoconcentration with reference to resting condition. This correction takes into account the effect of potential variations in plasma volume after exercise.

### 2.5. Statistical Analysis

Conventional statistical methods were used to calculate mean, standard deviation (SD) and standard error of mean (SEM). Prior to the comparison of each dependent variable, the Gaussian distribution of the data was verified by the Shapiro–Wilk test. Paired Student t test was used to compare the total number of repetitions achieved without assistance during the training session. The concentrations of leukocytes, CK, MB, IL-6, GH, cortisol, IGF-1 and lactate were transformed to the natural logarithmic scale. This transformation was conducted as a remedial measure to produce symmetrical measurement distributions and to equalize measurement variability [29]. Logarithmic data were then analyzed via a two-way ANOVA with repeated measures on two factors (method and time). When significant main effect or interactions were found, a Tukey post hoc test was used to determine differences between selected mean values. Coefficients of determination, extracted from Pearson product–moment correlations, were calculated to determine the association between the change in peak lactate before and after the training session (delta value) with the changes in cortisol, GH and IL-6. For all comparisons, the statistical level of significance was set at *p* < 0.05. For clarity, the *p* values reported in the figures correspond to those of the post hoc tests only. Values are expressed as mean ± SD in the text and mean ± SEM in the figures.

## 3. Results

### 3.1. Workload During Training Session

Subjects achieved a greater number of repetitions (*p* < 0.001) without assistance with the 8 × 6 method than the 3/7 method, representing 99.7 ± 0.7% and 83.6 ± 6.1 of the total planned workload, respectively. For the 8 × 6 method, the total number of repetitions for all four exercises was 191.4 ± 1.5 out of a theoretical maximum number of 192 repetitions. Failure was only observed for arm exercises. For the 3/7 method, the total number of repetitions for the four exercises was 167.3 ± 12.3 out of a theoretical maximum number of 200 repetitions. The number of repetitions performed without assistance for the 3/7 method was 31.8 ± 6.9 in bench press, 35.7 ± 5.5 in seated cable rows, 44.2 ± 7.5 in leg press and 44.1 ± 4.8 in standing calf raise for a theoretical maximum number of 50 per exercise.

### 3.2. Marker of Metabolic Response

**Figure 2:**
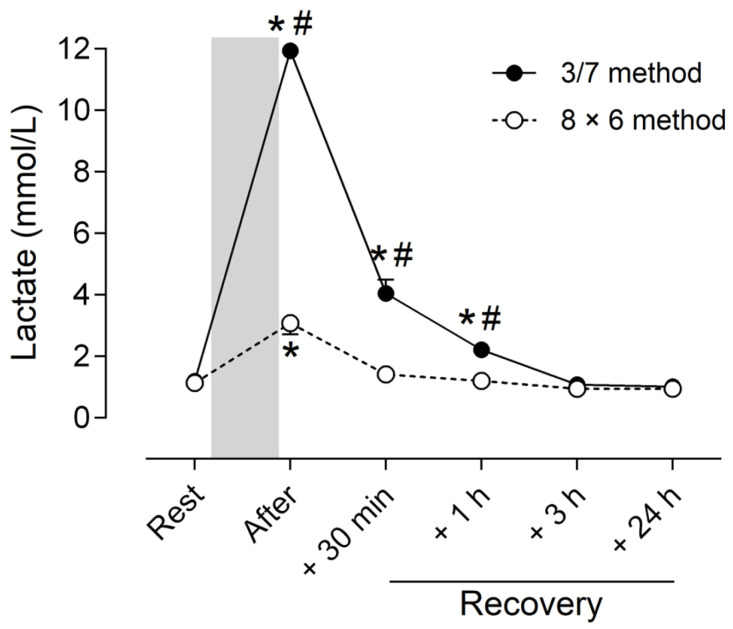
Blood lactate concentration before (Rest), immediately after the training session (After) and at 30 min (+30 min), 1 h (+1 h), 3 h (+3 h) and 24 h (+24 h) of recovery for the two strength training methods (3/7 method: filled circle; 8 × 6 method: open circle). The gray bar corresponds to the training session period. Post hoc tests indicate: * Significant difference (*p* < 0.05) with resting value for each method; # significant difference (*p* < 0.05) between methods at each time point.

The ANOVA showed a significant difference in blood lactate response between the two methods (method main effect, *p* < 0.001) and a significant difference with baseline (time main effect, *p* < 0.001), regardless of the training method. Moreover, a significant method × time interaction effect (*p* < 0.001) was observed for blood lactate concentration. Post hoc analyses revealed an increase (*p* < 0.001) in lactate concentration after the training session for both methods that remained above the control value (*p* < 0.001) at +30 min and +1 h of recovery but only for the 3/7 method (Figure 2). A greater increase (*p* < 0.001) was observed for the 3/7 method compared with the 8 × 6 method immediately and up to +1 h after the end of the training session (Figure 2).

### 3.3. Markers of Muscle Damage

**Figure 3:**
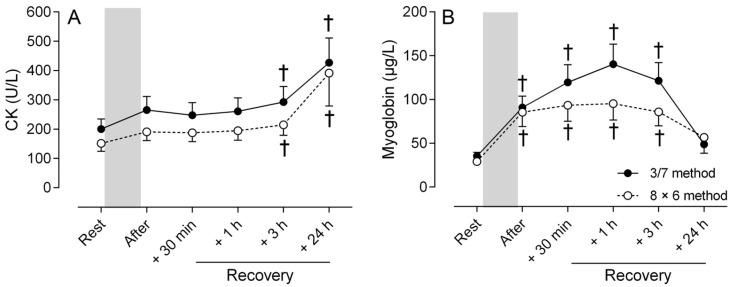
Concentrations of creatine kinase (CK, (**A**)) and myoglobin (**B**) before (Rest), immediately after the training session (After) and at 30 min (+30 min), 1 h (+1 h), 3 h (+3 h) and 24 h (+24 h) of recovery for the two strength training methods (3/7 method: filled circle; 8 × 6 method: open circle). The gray bar corresponds to the training session period. Post hoc tests indicate: † Significant difference (*p* < 0.05) with resting value, regardless of the training method.

Markers of muscle damage increased after the two training methods with no significant difference between methods (ANOVA, method main effect and method × time interaction, *p* > 0.05). Regardless of training method, CK concentration increased progressively after the training session (time main effect, *p* < 0.001), reaching significant differences with resting values at +3 h (post hoc test, *p* < 0.05) and +24 h (post hoc test, *p* < 0.001) following the training session (Figure 3A). Similarly, MB concentration increased after both training methods (ANOVA, time main effect, *p* < 0.001). Significant changes (post hoc test, *p* < 0.001) from resting value were observed immediately after the training session for the two methods, peaking at +1 h and remaining above the resting value up to +3 h before returning to the resting value at +24 h (Figure 3B).

### 3.4. Markers of Inflammatory Response

**Figure 4:**
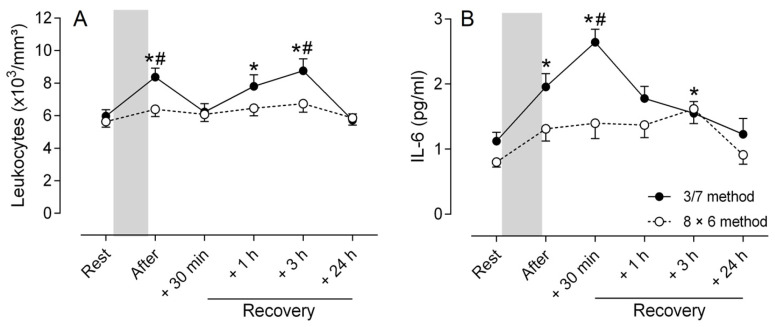
Concentrations of total leukocytes (**A**) and interleukine-6 (IL-6; (**B**)) before (Rest), immediately after the training session (After) and at 30 min (+30 min), 1 h (+1 h), 3 h (+3 h) and 24 h (+24 h) of recovery for the two strength training methods (3/7 method: filled circle; 8 × 6 method: open circle). The gray bar corresponds to the training session period. Post hoc tests indicate: * Significant difference (*p* < 0.05) with resting value for each method; # Significant difference (*p* < 0.05) between methods at each time point.

Markers of inflammatory response showed a significant difference between the two methods (ANOVA, method main effect, *p* < 0.05) and a significant difference with baseline regardless of the training method (ANOVA, time main effect, *p* < 0.001). A significant method × time interaction effect (*p* < 0.001) was observed in leukocytes concentration. Post hoc analyses revealed that a change was only observed for the 3/7 method and indicate rapid leukocytosis immediately (*p* < 0.001) after the training session (Figure 4A). Thereafter, leukocytes concentration returned to its resting value at +30 min after the last exercise and re-increased significantly (*p* < 0.001) at +1 h and +3 h of recovery before returning to the initial values at +24 h (Figure 4A).

For IL-6 concentration, the ANOVA indicated a significant method × time interaction effect (*p* < 0.01). Post hoc analyses revealed a significant increase (*p* < 0.01) immediately after the end of the training session, peaking at +30 min (*p* < 0.001), before returning progressively to the resting level for the 3/7 method (Figure 4B). In contrast, a slight increase (*p* < 0.01) in IL-6 concentration was only observed at +3 h of recovery for the 8 × 6 method (Figure 4B).

### 3.5. Markers of Hormonal Responses

The ANOVA showed a significant difference with baseline values for the markers of hormonal response regardless of the training method (time main effect, *p* < 0.01) and a significant method × time interaction effect (*p* < 0.05). However, only GH and cortisol responses showed a significant difference between the two methods (method main effect, *p* < 0.01).

**Figure 5:**
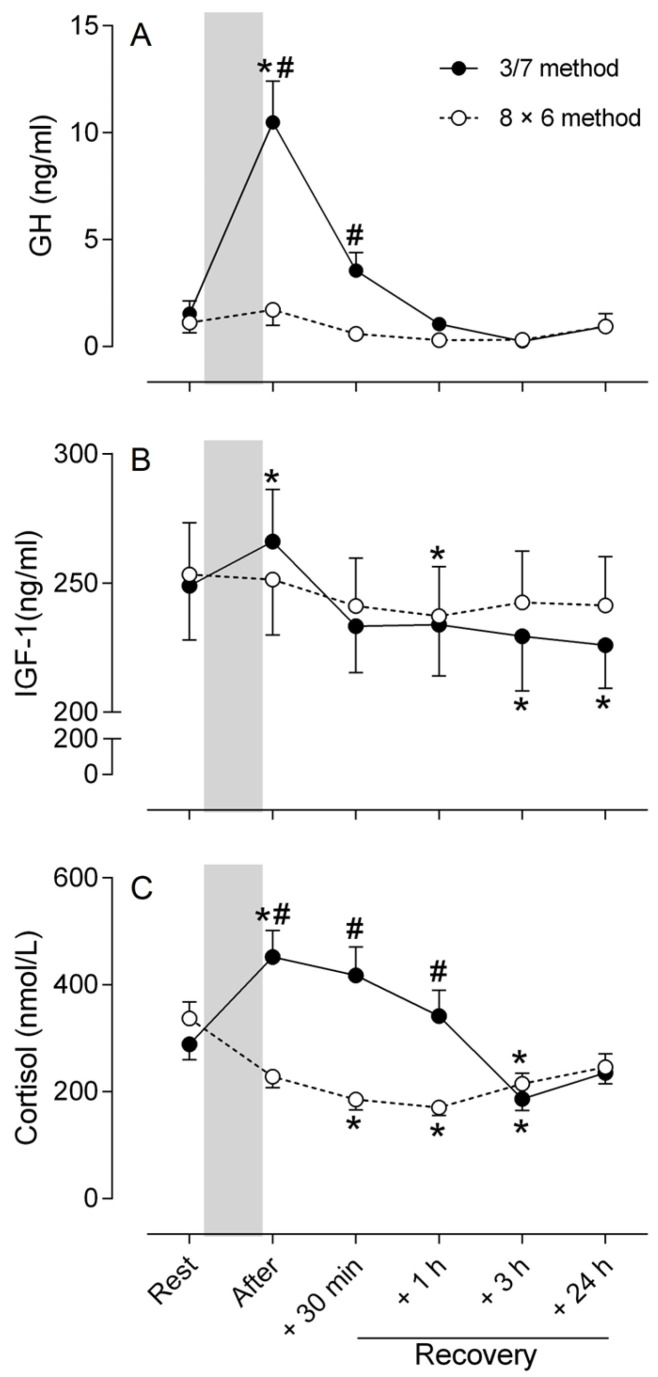
Concentrations of GH (**A**), IGF-1 (**B**) and cortisol (**C**) before (Rest), immediately after the training session (After) and at 30 min (+30 min), 1 h (+1 h), 3 h (+3 h) and 24 h (+24 h) of recovery for the two strength training methods (3/7 method: filled circle; 8 × 6 method: open circle). The gray bar corresponds to the training session period. Post hoc tests indicate: * Significant difference (*p* < 0.05) with resting value for each method; # Significant difference (*p* < 0.05) between methods at each time point.

A significant method × time interaction effect (*p* < 0.001) in GH concentration was observed with post hoc tests revealing an increase immediately (*p* < 0.001) after the training session for the 3/7 method, while no significant change was observed for the 8 × 6 method (Figure 5A). GH concentration returned to resting values from +1 h after the training session.

A significant method × time interaction effect for IGF-1 concentration was observed (*p* < 0.001). Post hoc analyses indicated an increase (*p* < 0.05) immediately after the end of the training session, returned to resting values at +30 min and +1 h of recovery for the 3/7 method before decreasing significantly below the control value at +3 h (*p* < 0.01) and +24 h (*p* < 0.001) (Figure 5B). In contrast, IGF-1 concentrations did not change after the training session for the 8 × 6 method, but the value was lower (post hoc test, *p* < 0.05) than the resting level at +1 h of recovery.

For cortisol concentration, the ANOVA indicated a significant method × time interaction effect (*p* < 0.001) (Figure 5C). Post hoc tests indicated that cortisol was augmented (*p* < 0.05) immediately after the training session for the 3/7 method, before decreasing progressively over time, reaching a blood concentration lower than the resting value at +3 h of recovery (Figure 5C). For the 8 × 6 method, cortisol concentration decreased after the training session, reaching its lowest values (*p* < 0.001) at +1 h of recovery (Figure 5C). For the two methods, cortisol concentration returned to resting values within 24 h of recovery.

### 3.6. Association Between Biomarkers

**Figure 6:**
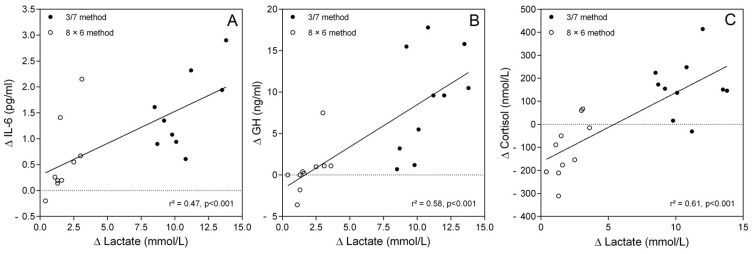
Associations between the increase in lactate concentration immediately after the training session relative to rest (∆ lactate) and the increase in IL-6 (∆ IL-6, (**A**)) before and 30 min after the training session or with the increase in GH (∆ GH, (**B**)) or in cortisol (∆ Cortisol, (**C**)), before and immediately after the training session. Data from the two methods (3/7 method: filled circle; 8 × 6 method: open circle) were pooled together.

Analyses showed positive associations between changes in some key biomarkers when data from the two training methods were pooled together (Figure 6). This is the case for the amount of changes in peak lactate before and immediately after the training session and those in peak IL-6 at +30 min (r^2^ = 0.47; *p* < 0.001; Figure 6A). Similarly, a positive association was found between the increase in peak lactate before and immediately after the training session and in peak GH (r^2^ = 0.58; *p* < 0.001; Figure 6B) or cortisol (r^2^ = 0.61; *p* < 0.001; Figure 6C).

## 4. Discussion

To our knowledge, this study is the first to combine the analysis of blood markers of metabolic, muscle damage, inflammatory and hormonal responses and their interaction in response to a training session of high-load exercises. The main findings of this study are greater metabolic, inflammatory and hormonal responses after the 3/7 method session that combines both high mechanical tension and metabolic stress compared with the 8 × 6 method characterized by a similar load but lower metabolic stress. The changes in key blood biomarkers provide an indication of the underlying factors that may potentially explain the greater efficacy of the 3/7 method observed in previous studies [25,26].

### 4.1. Workload Volume

Without any assistance, subjects performed less repetitions in the 3/7 method than the 8 × 6 method. Therefore, to match the training volume and to limit the influence of this confounding factor on the changes in blood markers, the experimenter provided, when needed, a minimal amount of assistance to the subject so that all sets were fully completed. As the load and the total number of repetitions were similar in the two training methods, it is suggested that the different responses were mainly influenced by the shorter recovery interval between sets and the organization of repetitions within the successive sets in the 3/7 method [3].

### 4.2. Metabolic Response

The greater difficulty to complete the total number of repetitions with the 3/7 method compared with the 8 × 6 method is likely due to the insufficient time to replenish the phosphocreatine (PCr) stores due to the reduced oxygen supply [27], as well as a greater fatigue-related accumulation of metabolites (lactate, H^+^, Pi, etc.) [11,12]. As expected, blood lactate concentration increased after the two strength training methods but to a greater extent for the 3/7 method than for the 8 × 6 method (11.9 ± 1.9 vs. 3.1 ± 1.1 mmol/L). Moreover, blood lactate remained above the control value up to 1 h post-training for the 3/7 method only. This finding is consistent with previous works using moderate intensity loading (60–85% of 1 RM) and volume (3–6 sets), and relatively short rest intervals (<90 s) between sets, showing that these methods may favor intracellular accumulation of venous blood lactate [30]. Although the peak lactate value is not extremely high after the 3/7 method, it nevertheless suggests a greater metabolic stress than for the 8 × 6 method. In that context, several studies have suggested that this metabolic stimulus may contribute to increase muscle mass and strength, either by a direct action as an anabolic signal [13,14,31,32], or indirectly through the intensification of muscle activation, by an increase in motor unit recruitment and discharge rate, to compensate for the fatigue-related loss of force [33]. Compared with the longer inter-set rest interval in the 8 × 6 method, the very brief rest period (15 s) between sets in the 3/7 method induced a greater metabolic stress in the involved muscles.

### 4.3. Muscle Damage

The increase in blood CK concentration is classically associated with muscle damage in response to high mechanical tension. Following high-load training, and in particular with eccentric contractions, CK response usually peaks between 24 to 96 h after the training session [34,35]. Even though CK was not tested later than 24 h post-exercise in our study, the increase was relatively modest compared with eccentric exercises [35,36].

Unlike CK, which shows great inter-individual variability, MB is a more sensitive marker, as it rises quickly after muscle damage [37]. Indeed, the increase in MB concentration is usually detectable immediately after the end of a training session comprising high-load exercises; however, there is still no agreement on the post-exercise time required to reach its peak value. Some authors observed a peak at +3 h [37] while others observed a peak at about +72 h [36], depending of the type of activity and its intensity. In the current study, MB increased immediately after the strength training session, peaking at +1 h, regardless of the training method. Previous studies have reported that post-exercise MB concentration correlates with neutrophil counts, especially in the delayed phase of leukocytosis [16]. As our analysis did not target the neutrophils, this observation cannot be confirmed, but it might be related to the increase in neutrophils as the peak value reached by MB occurs at approximately the same time as leukocytosis. As MB is released into the bloodstream with increasing muscle damage [37], we assume that they were relatively similar after the two training methods.

### 4.4. Inflammatory Response

A damaged muscle causes an invasion of inflammatory cells that play a role in the repair process during the recovery phase of a strenuous strength training session [16,38] but also contribute to the increase in muscle mass via the incorporation of satellite cells by muscle fibers [38]. Muscle satellite cells are the only known source to provide additional myonuclei and play a crucial role in muscle fiber regeneration and the growth of the muscle in response to strength training [39]. A training session of high-load exercises induces transient disturbances in immunity, including an increase in circulating leukocyte numbers and cytokine concentration post-exercise [18,40]. In our study, an increase in serum leukocytes was observed immediately after the 3/7 method that returned to the resting value at +30 min of recovery, before increasing again between 1 h and 3 h after the training session. In contrast, no change was observed for the 8 × 6 method. The particular time course of change in leukocytes in the 3/7 method could be explained by a different timing in the peak values of lymphocytes and neutrophils [18].

Although IL-6 is involved in the inflammatory processes, it is indirectly an anti-inflammatory factor. Indeed, IL-6, produced by the muscle during exercise, decreases the expression of pro-inflammatory cytokines [41] and increases the expression of anti-inflammatory cytokines, whilst it also induces neutrophil mobilization and activation [42]. The duration of the exercise seems to be a major factor in the magnitude of IL-6 increase [43]. It has also been reported that for the same training load (70% of 1 RM), an inter-set interval of 90 s results in a higher IL-6 response than an interval of 30 s, likely due to the ability of the subject to perform a higher volume of repetitions [17]. In contrast, our results demonstrated an increase in IL-6 that peaked at +30 min post-training for the 3/7 method without any change for the 8 × 6 method. Therefore, given the similar load and number of repetitions performed in both training methods, we assume that the metabolic stress induced by the 3/7 method influenced IL-6 production as suggested by the positive association (r^2^ = 0.47; *p* < 0.001) between its increase and the change in peak lactate concentration. These results agree with the proposal that exercise-induced metabolic stress might stimulate IL-6 release from the intracellular stores [44]. A more recent study supports this hypothesis by showing that IL-6 release during strenuous exercise is associated (r^2^ = 0.41) with local intramuscular lactate production despite that a causal link has not yet been demonstrated between these two factors [21].

### 4.5. Hormonal Response

During the recovery period following a strength training session, muscle tissue remodeling is initiated by a catabolism process which is followed by an anabolism process resulting in growth and repair [5]. Therefore, both catabolic and anabolic hormones play a role in stimulating the anabolic processes [5]. Moreover, the brief rest interval between sets in the 3/7 method should be an important factor for hormonal responses to strength training [45].

The increase in blood hormone concentrations after a strength training session with a brief inter-set rest interval is associated with increased metabolic stress [1]. Therefore, given the greater increase in blood lactate immediately after the session with the 3/7 method, it is not surprising to observe a greater increase in GH compared with the 8 × 6 method. As already mentioned in several studies [31,46], a positive association between lactate and GH secretion has been reported without ensuring a causal link. The impact of GH on muscle mass development is currently unclear as its direct action on muscle hypertrophy seems negligible [1]. Some studies indicate, however, that the hypertrophic effects of GH and circulating IGF-1 (primarily from hepatic origin) are complementary [1,47]. GH is thought to increase the net muscle protein synthesis indirectly by facilitating amino acid transport and availability via both endocrine and locally produced IGF-1 [48]. However, it seems unlikely that the increase in circulating IGF-1 immediately after the 3/7 method is explained by the increase in GH, as the response of the circulating IGF-1 to GH release is delayed (3–9 h) and its peak value is only reached 16–28 h after the end of the training session [5]. The increase in circulating IGF-1 concentration could be stimulated by a higher mechanical loading, suggesting greater muscle damage [49] or metabolite accumulation [24,30]. The greater blood lactate accumulation in the 3/7 method, without a significant difference in the changes in markers of muscle damage between our two training methods, supports the second alternative.

With respect to exercise, cortisol has primarily catabolic functions but plays also an important regulatory role in metabolic responses [5,50]. In muscle tissue, cortisol facilitates the initiation of protein breakdown, resulting in increased release of amino acids into the blood circulation. This mechanism can contribute to protein synthesis and the regulation of protein turnover during the recovery from exercise [51]. Interestingly, the 3/7 method resulted in a greater increase in cortisol immediately after the strength training session than the 8 × 6 method. While it remained higher than the control value up to 1 h of recovery for the 3/7 method, a decrease was observed from 30 min to 3 h after the training session. Strength training protocols with high metabolic demand (high volume, moderate to high load, brief inter-set interval) are known to induce a more pronounced increase in cortisol level. Moreover, in the study of Kraemer and Ratamess [5], a significant correlation between the increase in lactate and cortisol was reported. While the decrease observed after the exercise session for the 8 × 6 method can be surprising at first, its response appears to match the circadian rhythm profile of cortisol. Indeed, the peak cortisol level (approximately 399 nmol/L) is reached at ~08:30 before slowly decreasing thereafter [52], which suggests that the 8 × 6 session would have marginally influenced cortisol production.

### 4.6. Potential Influence of Lactate in Muscle Adaptative Responses to Strength Training

It is often reported [3,26,30,31,45] suggest that high-load exercise using brief rest intervals between sets might provide a superior stimulus for muscle hypertrophy than longer rest periods. The reason for this greater muscle adaptation may involve, in addition to mechanotransduction and satellite cell-related mechanisms, the contribution of metabolic, damage, inflammatory and endocrine factors.

The absence of a difference in the amount of changes in CK and MB between our two training methods suggests that muscle damage should have only a minor influence in the differences in training adaptations previously observed between the 3/7 method and the 8 × 6 method [26]. On the other hand, lactate response seems to play a substantial role in the superiority of the 3/7 method by augmenting the magnitude of the inflammatory and hormonal responses. Lactate response may influence many signaling cascades, including inflammatory factors, growth factors and hormone release [4,38]. In their recent review article, Lawson and colleagues [4] provide evidence from human studies for a potential role of lactate accumulation in muscle hypertrophy, in addition to mechanical stress. Moreover, studies performed in animals, in both in vitro and in vivo conditions, indicate that lactate is a potential anabolic molecule for muscle hypertrophy [13,14,32,53]. For example, these studies indicated that high lactate concentration can modulate muscle differentiation by regulating the associated networks of myogenic protein determination, enhancing thereby fast-twitch-type myosin heavy chain (MHC) expression and myotube hypertrophy in vitro [53]. Furthermore, Ohno and colleagues [54] reported that oral administration of lactate to mice increased muscle weight and protein content; these adaptations being accompanied by an elevated activation of G protein-coupled receptor 81 (GPR81), a selective lactate receptor and related extracellular signal-regulated kinase-1/2-pathway (ERK1/2) (see Figure 2 [4]). Therefore, the greater efficacy of the 3/7 method may be the consequence of a greater metabolic response. In addition, the production of IL-6, likely stimulated by the accumulation of metabolites, could influence muscle repair and hypertrophy by promoting both the proliferation of satellite cells and myogenic differentiation [22,55]. Despite that its precise effect on muscle hypertrophy is currently unknown, GH production, possibly influenced by lactate response, might indirectly increase the net synthesis of muscle proteins by facilitating the transport and availability of amino acids through endocrine and locally produced IGF-1 [48]. Furthermore, despite the limited understanding of lactate signaling and its impact on gene expression, recent studies indicate that lactate can induce post-translational modification named lysine lactylation (Kla), which may play a role in modulating the immune response and maintaining homeostasis [56]. Kla was found to be closely related to exercise, inducing a shift in microglial phenotype from pro-inflammatory to reparative lactylation in mice [57]. Additional mechanistic studies are, however, needed to substantiate this analysis and, as the evoked mechanisms may not have an immediate beneficial effect on muscle growth, to explore whether chronic exposure to elevated lactate concentration is an additive factor to high mechanical stress for muscle hypertrophy over time.

### 4.7. Study Limitations

Some methodological aspects of this study deserve mention. First, the relatively small sample size (*n* = 10) may have influenced the statistical power for some biomarkers and potentially limit the generalizability of the results. Indeed, it is known that responses to exercise-induced muscle damage vary among individuals depending on genetic background [58]. Second, the peak of CK was likely not reached at 24 h after the training session in the current study, which could have masked a possible delayed difference between the two methods. The return of MB values to baseline after only 24 h, however, suggests that our two strength training methods with a moderate eccentric component resulted in little muscle damage, which did not differ significantly between both methods. Third, an additional limitation concerns the comparison of two methods differing in the organization of sets (incremental vs. fixed number of repetitions), which could have biased the conclusions. In fact, our primary objective was to compare two strength training methods that induce very different metabolic stresses despite a similar total volume (number of repetitions) and load intensity (70% of 1 RM). In a previous study (27), we observed that beyond this slight difference in the organization of sets between these two methods, the metabolic demand quantified by the reduction in tissue oxygenation recorded by near-infrared spectroscopy (NIRS) was substantially greater for the 3/7 method compared with 4 × 6 and 8 × 6 methods. This study further indicated a clear cumulative reduction in tissue oxygenation over the successive sets of the 3/7 method due to the short inter-set intervals (15 s) but not for the longer inter-set intervals (2.5 min) of the 4 × 6 and 8 × 6 methods. We are therefore confident that the greater lactate production (metabolic stress) induced by the 3/7 method in the current study is primarily due to the very short rest duration between sets.

## 5. Conclusions

This study indicates that a strength training session with high load and very short inter-set intervals (15 s; 3/7 method) is associated with greater accumulation of lactate (metabolic stress) and enhanced inflammatory and hormonal responses compared to a method (8 × 6) with a similar load intensity and total volume but longer inter-set intervals (2.5 min). Together, these observations support the idea [1,4] that a training program combining high load (~70% of 1 RM) and a brief resting period (<30 s) between sets might provide a greater stimulus for muscle adaptation than mechanical tension alone.

## Data Availability

The data collected and analyzed during the current study are available from the corresponding author (A.C.) upon reasonable request.

## Abbreviations

The following abbreviations are used in this manuscript:

CK	creatine kinase
MB	myoglobin
IL-6	interleukine-6
GH	growth hormone
IGF-1	insulin-like growth factor-1
1 RM	one repetition maximal
H^+^	hydrogen ions
Pi	inorganic phosphate
3RM	3-repetitions maximum
PCr	phosphocreatine
MHC	myosin heavy chain
GPR81	G protein-coupled receptor 81
ERK1/2	extracellular signal-regulated kinase-1/2
Kla	lysine lactylation
